# circRNA_8521 promotes Senecavirus A infection by sponging miRNA-324 to regulate *LC3A*

**DOI:** 10.1186/s13567-024-01291-0

**Published:** 2024-04-05

**Authors:** Xiwang Yang, Rui Liu, Yunsha Du, Caiqiu Mei, Guangneng Zhang, Chen Wang, Yijun Yang, Zhiwen Xu, Wenting Li, Xiao Liu

**Affiliations:** 1 Southwest University, College of Veterinary Medicine, Chongqing, 400715 China; 2 Department of Infectious and Tropical Diseases, The Second Affiliated Hospital of Hainan Medical University, Haikou, 570100 China; 3 Ya’an People’s Hospital, Ya’an, 625000 China; 4 School of Public Health, Southern Medical University, Guangzhou, 511495 China; 5 Animal Biotechnology Center, College of Veterinary Medicine, Sichuan Agricultural University, Chengdu, 610052 China; 6 Department of Infectious Diseases, The First Affiliated Hospital of Anhui Medical University, Hefei, 230022 China; 7 State Key Laboratory of Silkworm Genome Biology, Chongqing, 400715 China

**Keywords:** Senecavirus A, circRNA, miRNA, LC3A

## Abstract

Senecavirus A (SVA) causes outbreaks of vesicular disease in pigs, which imposes a considerable economic burden on the pork industry. As current SVA prevention measures are ineffective, new strategies for controlling SVA are urgently needed. Circular (circ)RNA is a newly characterized class of widely expressed, endogenous regulatory RNAs, which have been implicated in viral infection; however, whether circRNAs regulate SVA infection remains unknown. To investigate the influence of circRNAs on SVA infection in porcine kidney 15 (PK-15) cells, RNA sequencing technology was used to analyze the circRNA expression profiles of SVA-infected and uninfected PK-15 cells, the interactions between circRNAs, miRNAs, and mRNAs potentially implicated in SVA infection were predicted using bioinformatics tools. The prediction accuracy was verified using quantitative real-time (qRT)-PCR, Western blotting, as well as dual-luciferase reporter and RNA pull-down assays. The results showed that 67 circRNAs were differentially expressed as a result of SVA infection. We found that circ_8521 was significantly upregulated in SVA-infected PK-15 cells and promoted SVA infection. circ_8521 interacted with miR-324. miR-324 bound to *LC3A* mRNA which inhibited the expression of LC3A. Knockdown of LC3A inhibited SVA infection. However, circ_8521 promoted the expression of LC3A by binding to miR-324, thereby promoting SVA infection. We demonstrated that circ_8521 functioned as an endogenous miR-324 sponge to sequester miR-324, which promoted LC3A expression and ultimately SVA infection.

**Supplementary Information:**

The online version contains supplementary material available at 10.1186/s13567-024-01291-0.

## Introduction

Senecavirus A (SVA) is the only member of the genus *Senecavirus* within the family *Picornaviridae* [1]; it is a non-enveloped, single-stranded RNA virus, which primary infects pigs [2]. The SVA genome spans ~7.3 kb and encodes a single polyprotein, which consists of the leader (L) and three major protein (P1, P2, and P3) regions; this polyprotein is subsequently processed into four structural proteins (VP1, VP2, VP3, and VP4) and eight nonstructural proteins (L, 2A, 2B, 2C, 3A, 3B, 3C, and 3D) by proteases 2A and 3C [3, 4]. SVA infection can cause severe vesicular and ulcerative lesions in the oral mucosa, snout, coronary bands, and hooves of pigs, which are indistinguishable from the clinical symptoms of infections caused by foot-and-mouth disease virus (FMDV), vesicular stomatitis virus (VSV), swine vesicular disease virus (SVDV), or and vesicular exanthema of swine virus (VESV); collectively, these viruses inflict serious losses on the pork industry [5–9]. SVA was accidentally discovered in 2002 in PER.C6 cell line culture sample [1]; however, SVA infection in pigs was not documented until 2007, when the first case was reported in North America. At the end of 2014/beginning of 2015, a sudden large-scale outbreak of SVA, associated with vesicular disease in pigs, was reported in Brazil [10, 11] and was subsequently reported in the United States [12], Canada [13], Colombia [14], China [15], Thailand [16], and Vietnam [17]. Recurrent outbreaks and global epidemics of SVA associated with vesicular disease have caused heavy economic losses and have threatened the pork industry.

SVA modulates many host cellular pathways, including the innate immune response, apoptosis, and autophagy. Innate immunity is the first line of host defense against viral infection. Upon infection, viruses are recognized by the host’s pattern recognition receptors (PRRs), leading to the activation of specific signaling cascades and the subsequent production of interferons (IFNs), which ultimately restrict viral replication and attenuate infection [18]. Increasing evidence suggests that SVA employs several cunning mechanisms to evade the host’s antiviral defenses to establish infection. For example, the 2AB protein of SVA promotes membrane associated ring-CH-type finger 8 (MARCHF8) and mitochondrial antiviral signaling protein (MAVS) degradation to inhibit IFN type I signaling [19]. SVA-induced glycolysis facilitates viral replication by promoting lactate production, which attenuates the interaction between MAVS and retinoic acid-inducible gene I [20]. The SVA 3C protein cleaves nuclear factor-kappa B (NF-κB) transcription factors NF-κB-p65, thereby promoting SVA replication and release [21]. SVA suppresses antiviral IFN production to escape from the host’s innate immune response by cleaving the host adaptor proteins MAVS, Toll/interleukin 1 receptor domain-containing adaptor inducing IFN-β, and tumor necrosis factor receptor-associated factor family member-associated NF-κB activator [22]. Besides, studies have shown that SVA participates in autophagy regulation. Autophagy is an evolutionarily conserved degradative process, which maintains host health by facilitating the capture and clearance of invading pathogens by the immune system [23, 24]. Autophagy is often initiated to curtail infection by delivering viral particles for lysosomal degradation and working alongside innate PRRs to induce IFN-mediated viral clearance. Although autophagy plays a crucial role in viral clearance, some viruses have evolved a variety of strategies to inhibit, evade, or manipulate multiple steps of this process to further their survival and propagation [25]. For instance, the accumulation of double-membrane vesicles has been reported following picornaviral infection. These small RNA viruses, such as Coxsackievirus B3 [26], hepatitis C virus [27, 28], FMDV [8], and Zika virus [29], can use autophagosome membranes for RNA assembly and self-replication. Similarly, SVA can hijack autophagic machinery to replicate [30]. Apoptosis can be used as a host defense mechanism to kill virally-infected cells. However, several picornaviruses, including SVA, have been shown to induce apoptosis to promote viral transmission while avoiding host inflammatory responses and immune system activation. For instance, the 3C protease of SVA, which plays a critical role in the viral infection cycle, induces host cell apoptosis to facilitate viral release from infected cells [21].

Circular (circ)RNAs are covalently closed, endogenous biomolecules, which can be produced from viral RNA genomes or the processing of cellular housekeeping noncoding (nc)RNAs and precursor messenger (m)RNAs [31]. In eukaryotes, circRNAs are abundant, evolutionarily conserved, and exhibit tissue/cell-specific expression patterns. circRNAs exert biological functions by acting as transcriptional regulators, microRNA (miRNA) sponges, and protein templates, scaffolds, or recruiters [32]. The distinct expression signatures and important biological roles of circRNAs have been documented in a variety of diseases. In the context of viral infections, circRNAs are implicated in the control of miRNA levels and the regulation of innate immunity. circRNAs are also involved in the regulation of viral infections such as those caused by hepatitis B virus, human immunodeficiency virus, severe acute respiratory syndrome coronavirus 2, and human herpesvirus [33]. In addition, circRNAs have been used as biomarkers to distinguish viral from non-viral pneumonia [34]. However, whether and how circRNAs regulate SVA infection is largely unknown.

In this study, we identified that circ_8521, originating from the AGO3 gene locus (exons 7–11), was upregulated in the porcine kidney 15 (PK-15) cell line following SAV infection. Functional assays showed that circ_8521 promoted SAV infection. Mechanistically, circ_8521 functioned as a competing endogenous circRNA by sponging miR-324 in PK-15 cells to upregulate the expression of the gene encoding microtubule associated protein 1 light chain 3 alpha (LC3A), thereby facilitating SAV infection.

## Materials and methods

### Cell culture and viral infection

PK-15 cell was obtained from the Cell Resource Center, Peking Union Medical College (Beijing, China) were cultured in DMEM (Thermo Fisher HyClone, Waltham, MA, USA) supplemented with 10% fetal bovine serum (GIBCO, Waltham, MA, USA) at 37 °C under 5% CO_2_ in a humidified incubator. Primary porcine nasal mucosal epithelial cell was purchased from SAIOS (Wuhan, China) were cultured in primary epithelial cell medium (SAIOS, Wuhan, China) at 37 °C under 5% CO_2_ in a humidified incubator. Intestinal porcine epithelial cell was purchased from SAIOS (Wuhan, China) were cultured in DMEM (Thermo Fisher HyClone, Waltham, MA, USA) supplemented with 5% fetal bovine serum (GIBCO, Waltham, MA, USA) at 37 °C under 5% CO_2_ in a humidified incubator. SVA-CQ strain (GenBank accession no. OP696593) was used in this study. SVA was propagated in PK-15 cells, whereby the cells were infected with the virus at a multiplicity of infection (MOI) of 2.

### Antibodies

The anti-SVA rabbit monoclonal antibody (Sangon Biotech, Shanghai, China), anti-SQSTM1/p62 rabbit monoclonal antibody (Beyotime, Shanghai, China), anti-LC3A/MAP1LC3A rabbit monoclonal antibody (Beyotime, Shanghai, China), anti-β-actin mouse monoclonal antibody (Cell Signaling, Danvers, MA, USA), anti-LC3B rabbit polyclonal antibody (Beyotime, Shanghai, China), horseradish peroxidase (HRP)-conjugated goat anti-rabbit IgG (Cell Signaling, Danvers, MA, USA), and HRP-conjugated goat anti-mouse IgG (Cell Signaling, Danvers, MA, USA) were used in this study.

### Cyclization validation

The cyclization of circ_8521 was validated using RNase R (Beyotime, Shanghai, China) by following the manufacturer’s instructions. Briefly, ~4 µg of isolated RNA was incubated with RNase R (3 U/µg RNA) for 30 min at 37 °C, which was subsequently deactivated at 70 ℃ for 10 min. Finally, quantitative real-time (qRT)-PCR analysis was performed.

### Subcellular fractionation

PK-15 cells were washed with PBS. The nuclear and cytoplasmic fractions were then isolated using the Nuclear and Cytoplasmic Extraction Kit (Beyotime, Shanghai, China) according to the manufacturer’s protocol. *GAPDH* and *U6* small nuclear RNA were used as the expression standards in the cytoplasmic and nuclear fractions, respectively.

### RNA extraction and qRT-PCR

Total RNA was isolated using the Tiangen Total RNA Isolation Kit (Tiangen, Beijing, China) by following the manufacturer’s instructions. The concentration of RNA was measured on a NanoDrop ND-2000 spectrophotometer (Thermo Scientific NanoDrop, DE, USA), and the RNA was reverse transcribed into cDNA using the Plus All-in-one Strand cDNA Synthesis SuperMix (Novoprotein, Shanghai, China). qRT-PCR was performed on a Bio-Rad iQ5 system (Bio-Rad, Shanghai, China) using the NovoStart SYBR qPCR superMix Plus kit (Novoprotein, Shanghai, China). *GAPDH* and *U6* were used as internal controls for normalization of expression. The results were calculated using the 2^−ΔΔCt^ method. Primers are listed in the Additional file 1.

### Plasmids, oligonucleotides, and transfection

Circ_8521 overexpression plasmid pEZX-circ_8521 were constructed by GeneCopoeia (Guangzhou, China). Two circ_8521 siRNAs targeting different portions of the circ_8521 sequences were used. The sequences of the sense strands of the siRNA duplexes were as follows: si-circ_8521-1: 5′-CCAGCCUUGCUUCUGCAAATT-3′; si-circ_8521-2: 5′-GCCAGUGUAUGGUAAGGAUTT-3′. Two *LC3A* siRNAs targeting different portions of the *LC3A* sequence were produced. The sequences of the sense strands of the siRNA duplexes were as follows: si-*LC3A*-1: 5′-GGCUUCCUCUACAUGGUCUTT-3′; si-*LC3A*-2: 5′-GGAAACCUUCGGCUUCUGATT-3′. A non-targeting siRNA with the following sequence was used as the negative control (NC): 5′-UUCUCCGAACGUGUCACGUTT-3′. An miR-324 mimic, an miR-324 inhibitor, non-targeted random sequences, an miR-324 with biotin at the 3' end (biotin-miR-324; 5′-CGCAUCCCCUAGGGCAUUGGUGU-3′), and a biotinylated control miRNA (biotin-miR-NC; 5′-UUGUACUACACAAAAGUACUG-3′) were also generated. All the above sequence were designed and synthesized by Sangon Biotech (Shanghai, China). All transfections were performed according to the manufacturer’s specifications. The plasmids and oligos were transfected into PK-15 cells using the X-tremeGENE HP DNA Transfection Reagent (Roche, Basel, Switzerland).

### Dual-luciferase reporter assays

The *LC3A* sequence targeted by miR-324 was inserted into the pEZX luciferase reporter to establish the wild-type vector (pEZX-LC3A-WT). Next, the mutated (MUT) version of the seed sequence was designed to generate the mutated vector (pEZX-LC3A-MUT). pEZX-LC3A-WT and pEZX-LC3A-MUT were constructed by GeneCopoeia (Guangzhou, China). Recombinant vectors (500 ng) and miR-324 mimic or mimic-NC (50 pmol) were subsequently transfected into PK-15 cells; the luciferase activity was measured 48 h later using the Dual Luciferase Reporter Gene Assay Kit (Beyotime, Shanghai, China) as per the manufacturer’s protocol. Levels of *firefly* luciferase activity were normalized to that of *Renilla* luciferase.

### RNA pull-down assay

PK-15 cells were seeded into duplicate 10-cm tissue culture dishes, before being transfected with biotin-miR-NC or biotin-miR-324 at a final concentration of 60 nM, according to manufacturer’s instructions. Cells were harvested at 48 h post-transfection to generate whole cell lysates. The Streptavidin Magnetic Beads (Beyotime, Shanghai, China) were coated with yeast tRNA in advance to prevent nonspecific RNA binding. The cell lysates were then incubated with the beads at 4 ℃ for 12 h to isolate the RNA. The RNA was then precipitated using the standard chloroform-isopropanol method. Finally, the enrichment of circ_8521 was evaluated by qRT-PCR.

### Western blotting

PK-15 cells were washed twice with cold PBS and the total protein was extracted from cells with RIPA lysis buffer (Sigma, St. Louis, MO, USA). The protein concentration within the cell lysate was measured using the Bradford Protein Assay Kit (Beyotime, Shanghai, China). Equal amounts of protein were separated by sodium dodecyl sulfate polyacrylamide gel electrophoresis (SDS-PAGE) and the protein bands were transferred onto PVDF membranes (Merck Millipore, Billerica, MA, USA). After blocking with 5% bovine serum albumin (BSA) for 60 min at room temperature, the membranes were incubated with specific primary antibodies at 4 °C overnight, followed by HRP-conjugated secondary antibodies at room temperature for 1 h. β-actin was used as an internal control of protein expression. Finally, the bands were detected using the ChemiDoc MP Imaging System (Bio-Rad, Shanghai, China).

### Cell viability assay

Cell viability was determined using Cell Counting Kit-8 (CCK-8; Beyotime, Shanghai, China). Briefly, PK-15 cells were pre-seeded into 96-well plates, before being exposed to various transfectants for 24 h. The cells were then incubated with CCK-8 solution (10 µL/well) for 0.5–4 h. Finally, the absorbance was measured at 450 nm.

### Statistical analysis

Each experiment was repeated three times. All the data were expressed as the mean ± standard error of the mean. The Student’s *t*-test was used to determine the differences between two experimental groups, whereby *P*-values < 0.05 were used as a measure of statistical significance.

## Results

### Expression pattern of circRNAs changes upon SVA infection

**Figure 1:**
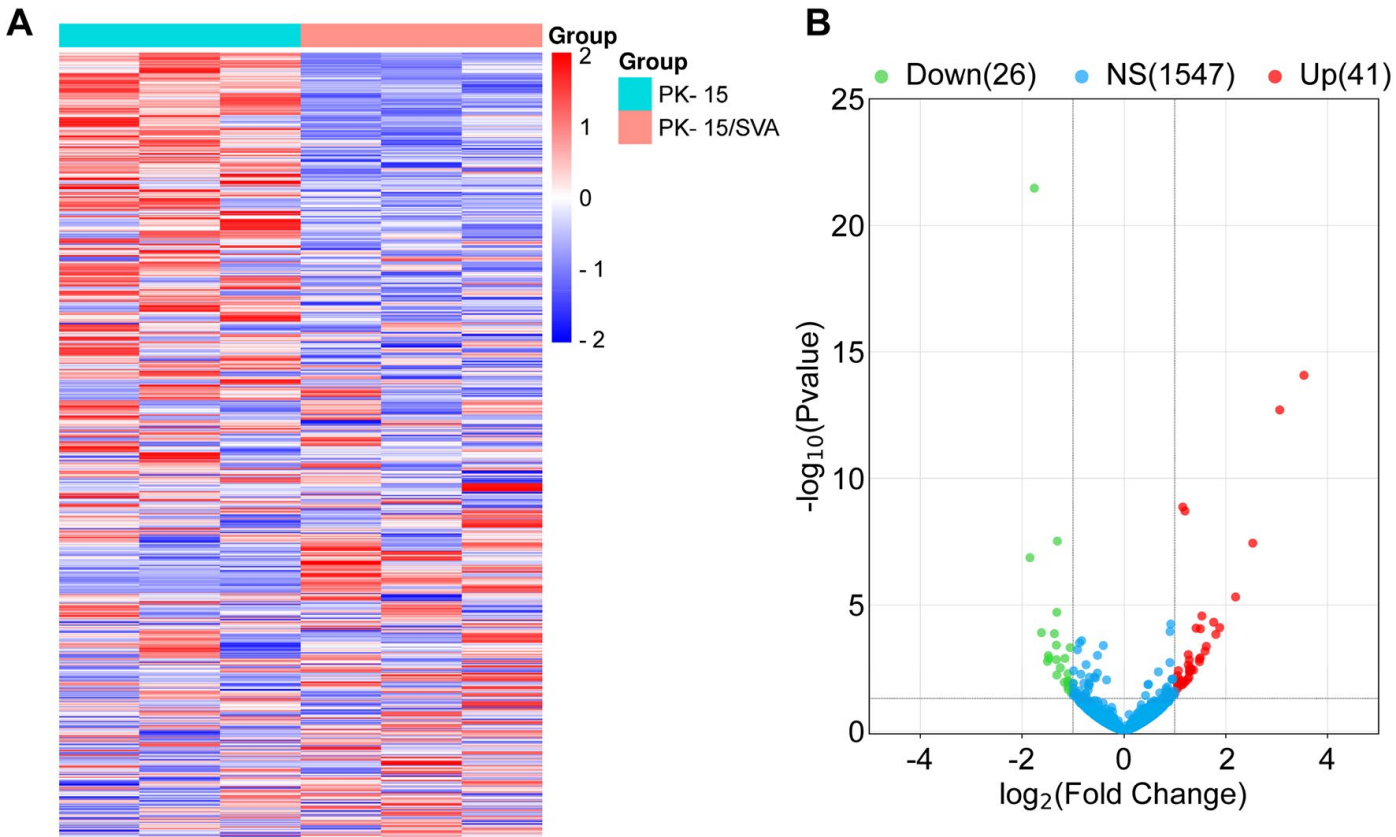
**The pattern of circRNA expression in PK-15 cells changes following SVA infection. A** Heatmap showing circRNAs expressed in uninfected or SVA-infected PK-15 cells. The red line indicates high relative expression, and the blue line indicates low relative expression. **B** Volcano map of differential circRNAs expression between uninfected and SVA-infected PK-15 cells. The cutoff values fold change > 2 and *P* < 0.05 were used to identify differentially expressed circRNAs. Red represents upregulated circRNAs, green represents downregulated circRNAs, and blue represents unchanged circRNAs.

To explore genome-wide circRNA expression during SVA infection, SVA-infected or uninfected PK-15 cells were subjected to RNA-seq. We found that SVA-infected PK-15 cells and control PK-15 cells had different circRNA expression patterns (Figure 1A). In total, 67 circRNAs were differentially expressed between SVA-infected and control PK-15 cells (fold difference ≥ 2 and *P* < 0 0.05), including 41 upregulated circRNAs and 26 downregulated circRNAs (Figure 1B). These findings indicate that SVA infection alters the circRNA expression pattern of host cells.

### Identification of circ_8521 as an SVA-inducible host circRNA

**Figure 2:**
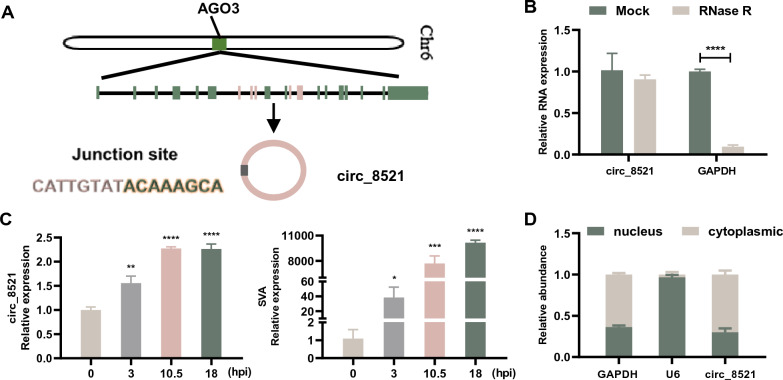
**Identification of circ_8521 as an SVA-inducible host circRNA. A** Schematic illustration showing that the genomic sequence of circ_8521 is derived from five exons (spanning 690 nt) of the *AGO3* gene. The sequences of the circ_8521 junction site are also shown. **B** qRT-PCR analysis of *GAPDH* RNA and circ_8521 levels after RNase R treatment. **C** The levels of circ_8521 (left) and SVA *VP1* mRNA (right) in PK-15 cells at 0, 3, 10.5, 18 h post-infection (hpi) were determined by qRT-PCR. **D** The levels of circ_8521 were assessed by qRT-PCR in nuclear and cytoplasmic fractions of the PK-15 cell lysate. *GAPDH* and *U6* served as the cytoplasmic and nuclear localization controls, respectively. *P < 0.05; **P < 0.01; ***P < 0.001; ****P < 0.0001.

Circ_8521 is a 690-nt circRNA derived from exons 7–11 of *AGO3* (Figure 2A). The circular structure of circ_8521 was confirmed in an RNase R treatment assay (Figure 2B). qRT-PCR analysis showed that circ_8521 had a cyclic structure, which made it resistant to RNase R treatment compared with the linear *GAPDH* control RNA. Next, we examined the expression dynamics of circ_8521 in more detail. A time course analysis of circ_8521 expression was performed using SVA-infected PK-15 cells. The results showed that the expression of circ_8521 was significantly increased in response to SVA infection (Figure 2C), Meanwhile, the upregulation of SVA mRNA expression confirmed active viral infection (Figure 2C). And SVA similarly induced upregulation of circ_8521 in primary SVA-targeted cells, such as primary porcine nasal mucosal epithelial cells and intestinal porcine epithelial cells (Additional file 2). Furthermore, we investigated the subcellular localization of circ_8521 as a first step toward understanding its functional role. The cytoplasmic *GAPDH* and nuclear *U6* transcripts were used as controls when determining the purity of cytoplasmic and nuclear fractions, respectively. The results showed that circ_8521 was predominantly localized to the cytoplasm (Figure 2D), suggesting that it might regulate gene expression at the post-transcriptional level. Together, these results demonstrate that the upregulation of circ_8521 is associated with SAV infection.

### circ_8521 regulates SVA infection in PK-15 cells

**Figure 3:**
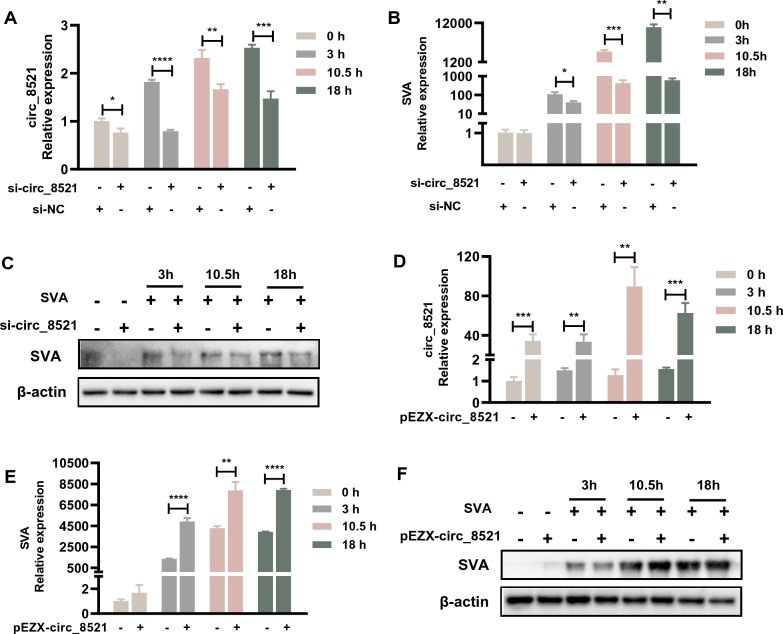
**circ_8521 regulated SVA infection in PK-15 cells.** PK-15 cells were transfected with circ_8521 siRNA (50 nM) (**A**–**C**) or the circ_8521 overexpression plasmid pEZX-circ_8521 (0.5 ng) (**D**–**F**). NC siRNA served as a control. At 24 h post-transfection, cells were infected with SVA for 3, 10.5, or 18 h. The effect of circ-8521 on SVA infection was subsequently analyzed. **A** qRT-PCR analysis circ_8521 levels. **B** qRT-PCR analysis SVA *VP1* mRNA levels. **C** Western blotting analysis of SVA protein expression. **D** qRT-PCR analysis of circ_8521 levels. **E** qRT-PCR analysis SVA *VP1* mRNA levels. **F** Western blotting analysis of SVA protein expression. *P < 0.05; **P < 0.01; ***P < 0.001; ****P < 0.0001.

To further assess the role of circ_8521 during SVA infection, PK-15 cells were transfected with circ_8521 siRNA. After confirming the knockdown of endogenous circ_8521 (Figure 3A), we showed that the transfection of circ_8521 siRNA significantly decreased SVA mRNA expression in PK-15 cells (Figure 3B). Western blotting confirmed that these changes also occurred at the protein level (Figure 3C). Conversely, overexpression of circ_8521 (Figure 3D) significantly increased SVA mRNA (Figure 3E) and protein (Figure 3F) levels. Cell viability is not affected by circ_8521 siRNA and pEZX-circ_8521 transfection (Additional file 3). These results demonstrate that the expression of circ_8521 facilitates SAV infection in PK-15 cells. In addition, we found that circ_8521 could also promote SVA infection in both primary porcine nasal mucosal epithelial cells (Additional file 4) and intestinal porcine epithelial cells (Additional file 5).

### circ_8521 promotes LC3A expression by binding to miR-324

**Figure 4:**
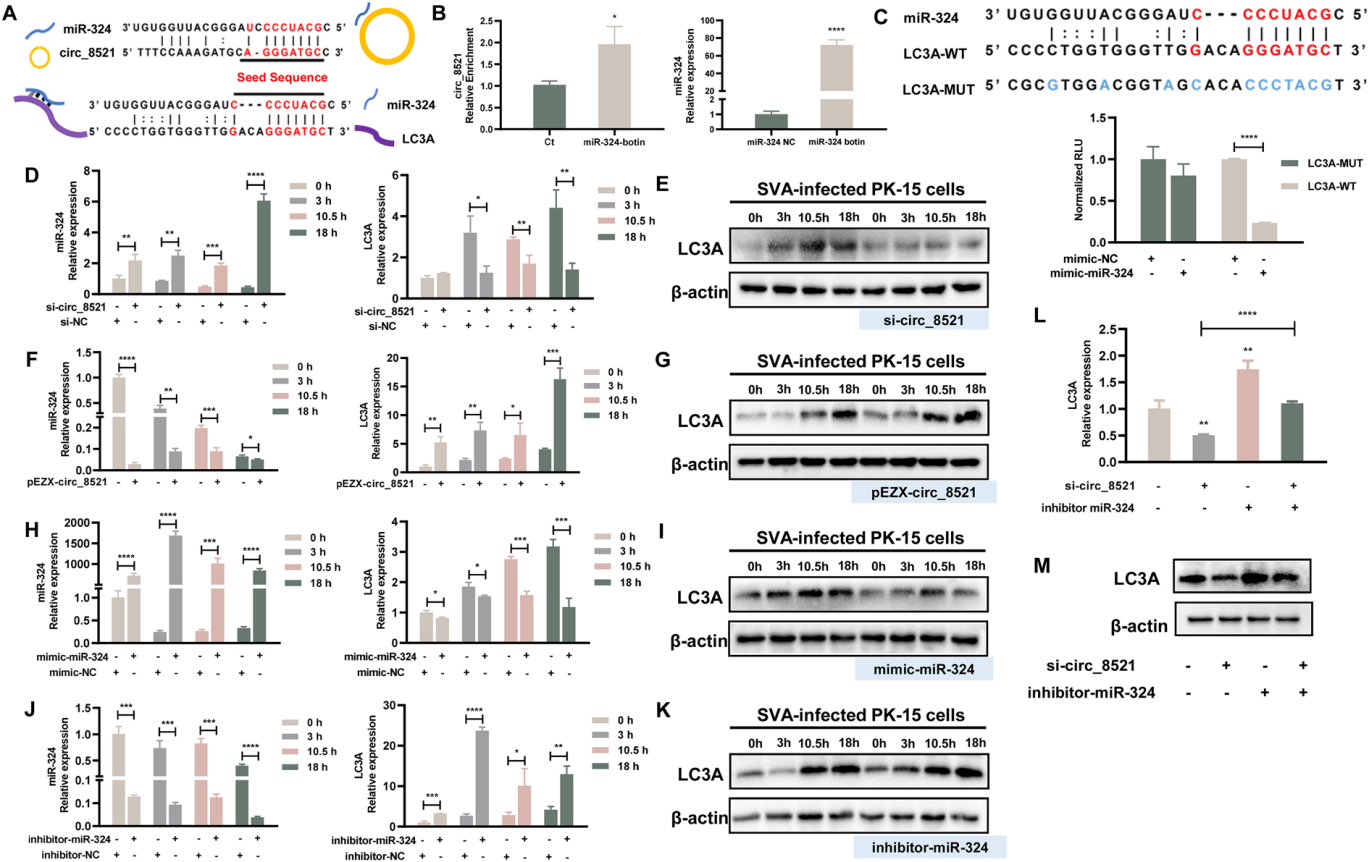
**circ_8521 promotes the expression of LC3A by binding to miR-324.** **A**Schematic diagram of the complementary sequences between circ_8521 and miR-324 and between miR-324 and *LC3A* mRNA. **B** PK-15 cells were transfected with biotin-coupled miR-324 mimic. Fold enrichment of circ_8521 was quantified by qRT-PCR after miRNA pull-down (left). qRT-PCR analysis of miR-324 levels was performed to determine transfection efficiency (right). **C** Schematic diagram showing the binding of miR-324 to wild-type *LC3A* (*LC3A*-WT) and mutant *LC3A* (*LC3A*-MUT) luciferase reporter vector (upper part). Detection of luciferase activity following the co-transfection of the luciferase reporter vector and miR-324 or control mimic into PK-15 cells for 48 h (lower part). *Firefly* luciferase activity was normalized to that of *Renilla* luciferase. PK-15 cells were transfected with circ_8521 siRNAs and NC siRNA served as control (**D**, **E**). PK-15 cells were transfected with pEZX-circ_8521 (**F**, **G**). PK-15 cells were transfected with the miR-324 mimic and NC mimic served as control (**H**, **I**). PK-15 cells were transfected with miR-324 inhibitor and NC inhibitor served as control (**J**, **K**). At 24 h post-transfection, cells were infected with SVA for 3, 10.5, or 18 h. **D** miR-324 (left) and *LC3A* mRNA (right) levels were detected by qRT-PCR. **E** LC3A protein was examined by Western blotting. **F** miR-324 (left) and *LC3A* mRNA (right) levels were detected by qRT-PCR. **G** LC3A protein was examined by Western blotting. **H** miR-324 (left) and *LC3A* mRNA (right) levels were detected by qRT-PCR. **I** LC3A protein was examined by Western blotting. **J** miR-324 (left) and *LC3A* mRNA (right) levels were detected by qRT-PCR. **K** LC3A protein was examined by Western blotting. PK-15 cells were co-transfected with miR-324 inhibitor and circ_8521 siRNAs. At 24 h post-transfection, cells were infected with SVA for 12 h. **L** *LC3A* mRNA levels were detected by qRT-PCR. **M** LC3A protein was examined by Western blotting. *P < 0.05; **P < 0.01; ***P < 0.001; ****P < 0.0001.

As a cytoplasmic RNA, circ_8521 has the potential to bind and sequester certain miRNAs in a sequence-specific manner, consequently repressing their function. We therefore used miRanda v3.3a [35], an algorithm used for the detection of potential microRNA target sites, to predict the putative binding partners of circ_8521. The results revealed that circ_8521 potentially interacted with miR-324. In addition, we used miRanda v3.3a to predict that miR-324 targeted *LC3A* transcripts. The highly conserved miR-324/circ_8521 and miR-324/*LC3A* binding sites are shown in Figure 4A. Given the interactions identified, we hypothesized that circ_8521 regulated SVA replication via the miR-324/*LC3A *axis. We used an RNA pull-down assay to confirm whether miR-324 indeed interacted with circ_8521. We observed the enrichment of circ_8521 in PK-15 cells transfected with the biotin-coupled miR-324 mimic compared with the negative control (Figure 4B). Next, the predicted pairing between miR-324 and *LC3A* mRNA was confirmed using a luciferase reporter assay with a vector containing WT or mutant (with no complementarity to miR-324) *LC3A* sequences (Figure 4C). The PK-15 cells co-transfected with the miR-324 mimic and *LC3A*-WT exhibited significantly lower luciferase activity than those in the *LC3A*-MUT group (Figure 4C), which confirmed the physical binding of miR-324 to *LC3A*.

Subsequently, we investigated the effect of circ_8521 on LC3A expression by sponging miR-324. The Western blotting and qRT-PCR results showed that the transfection of circ_8521 siRNA significantly increased miR-324 levels (Figure 4D) while reducing the expression of LC3A (Figures 4D, E). By contrast, overexpressing circ_8521 significantly reduced miR-324 levels (Figure 4F) and upregulated LC3A expression (Figures 4F, G). Transfecting PK-15 cells with the miR-324 mimic significantly increased miR-324 levels (Figure 4H) and downregulated the expression of LC3A (Figures 4H, I). Meanwhile, transfection with the miRNA inhibitor significantly knocked down miR-324 expression (Figure 4J) while increasing that of LC3A (Figures 4J, K). Cell viability is not affected by circ_8521 siRNA, pEZX-circ_8521, miR-324 mimic, and miR-324 inhibitor transfection (Additional file 3). Notably, when PK-15 cells were co-transfected with the miR-324 inhibitor and the circ_8521 siRNA, miR-324 knockdown partly rescued the circ_8521-ablation-induced downregulation of LC3A (Figures 4L, M). These results indicate that circ_8521 can function as an endogenous miR-324 sponge to promote LC3A expression.

### The circ_8521/miR-324/*LC3A* axis regulates SVA infection

**Figure 5:**
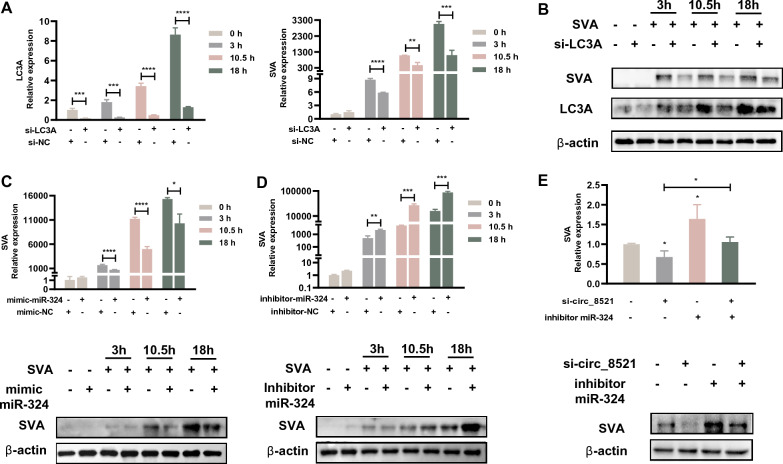
**The circ_8521/miR-324/*LC3A*** **axis regulates SVA infection**. PK-15 cells were transfected with *LC3A* siRNA. NC siRNA served as a control. At 24 h post-transfection, the cells were infected with SVA for 3, 10.5, or 18 h (**A**, **B**). **A** *LC3A* (left) and SVA *VP1* mRNA (right) levels were detected by qRT-PCR. **B** The expression of LC3A and SVA proteins were examined by Western blotting. **C** PK-15 cells were transfected with an miR-324 mimic. NC mimic served as a control. At 24 h post-transfection, the cells were infected with SVA for 3, 10.5, or 18 h. SVA *VP1* mRNA levels were detected by qRT-PCR (upper panel). SVA protein expression was examined by Western blotting (lower panel). **D**. PK-15 cells were transfected with an miR-324 inhibitor. NC inhibitor served as a control. At 24 h post-transfection, the cells were infected with SVA for 3, 10.5, or 18 h. SVA *VP1* mRNA levels were detected by qRT-PCR (upper panel). SVA protein expression was examined by Western blotting (lower panel). **E** PK-15 cells were co-transfected with an miR-324 inhibitor and circ_8521 siRNA. At 24 h post-transfection, the cells were infected with SVA for 12 h. SVA *VP1* mRNA levels were detected by qRT-PCR (upper panel). SVA protein expression was examined by Western blotting (lower panel). *P < 0.05; **P < 0.01; ***P < 0.001; ****P < 0.0001.

Given that circ_8521 facilitates SVA infection and is involved the regulation of LC3A expression, we next sought to explore the role of the circ_8521/miR-324/*LC3A* axis during SVA infection. We began by transfecting PK-15 cells with *LC3A* siRNA to determine whether LC3A promoted SVA infection; in these experiments, a random siRNA was used as a negative control. Knockdown of LC3A expression significantly decreased the extent of SVA infection, as determined by qPCR (Figure 5A) and Western blotting (Figure 5B). Cell viability is not affected by *LC3A* siRNA (Additional file 3). Next, PK-15 cells were transfected with an miR-324 mimic and a miR-324 inhibitor; again, a random RNA was used as a negative control. Knockdown of miR-324 significantly reduced the extent of SVA infection (Figure 5C), while overexpression of miR-324 had the opposite effect (Figure 5D). Finally, to further verify whether circ_8521 regulated the extent of SVA infection via miR-324, PK-15 cells were co-transfected with a miR-324 inhibitor and circ_8521 siRNA. We found that silencing of miR-324 in PK-15 cells partly rescued them from the effects of circ_8521 siRNA during SVA infection (Figure 5E). These results support the notion that the circ_8521/miR-324/*LC3A* axis regulates SVA infection.

## Discussion

In this study, we showed that during infection, SVA adjusted the level of citrc_8521 in the host cell to promote SVA infection through the miR-324/*LC3A* axis. Most (98%) of the mammalian genome is transcribed into ncRNAs. An increasing number of studies have found that ncRNAs play an important role in SVA infection and host antiviral immunity. For instance, the long ncRNA (lncRNA) lnc-MSTRG.18940.1 participates in host immunity and inflammation during SVA infection [36]. In PK-15 cells, SVA downregulates the expression of host lncRNA320, which is involved in the regulation of the TLR3 signaling pathway via the lncRNA320-sc-miR-7-CCR3 axis to promote IFN-β expression in response to SVA infection [37]. Moreover, SVA affects apoptosis by influencing the host's circadian rhythm through piRNA [38]. These findings provide important theoretical and practical guidance for SVA prevention and control, whereby the closed, circular structures of circRNAs may confer distinct advantages in disease diagnosis and as potential therapeutic targets. As a class of single-stranded RNAs with covalently-linked head-to-tail topology, circRNAs are less prone to degradation, which facilitates their detection and increases their utility [39]. Moreover, circRNAs have diverse functions. Recent research has revealed that circRNAs encoding certain polypeptides could be exploited in mRNA vaccine development [40, 41].

To the best of our knowledge, the present study is the first to describe the role of circRNAs in the regulation of SVA infection. However, the exact mechanisms of how SVA regulates ncRNAs such as circRNAs in the host cell is largely unknown. Revealing how viruses regulate the transcription of noncoding parts of the host genome and the biogenesis of ncRNAs such as circRNAs will allow us to gain a deeper understanding of the relationship between viruses, host immunity, and the pathogenesis of diseases; this important endeavor requires further research.

LC3A is an essential component of autophagosomes. Given that circ_8521 and miR-324 are upstream regulators of LC3A, we wondered whether they were implicated in autophagy. Therefore, we conducted a preliminary exploration on the effects of circ_8521 and miR-324 on autophagy in PK-15 cells during SVA infection (Additional file 6). We measured autophagy flux by detecting the levels of LC3-I, LC3-II, and p62. The results showed that after the cells were infected with SVA, their autophagy level increased. Moreover, knocking down circ_8521 or overexpressing miR-324 in SVA-infected cells reduced their autophagy levels. By contrast, knocking down miR-324 in SVA-infected cells increased their autophagy levels. Collectively, our results suggest that SVA increases autophagy levels in cells via the circ_8521/miR-324 axis to promote viral infection. Many studies have also shown that SVA can promote its replication through autophagy. For instance, Song et al. found that SVA activated the AKT/AMPK/MAPK/MTOR signaling pathway through the synergistic effect of its proteins VP1, VP3, and 3C, which led to the induction of complete autophagy and promoted viral replication [42]. Another study showed that SVA infection induced autophagy, which increased the SVA infection of pig cells [43]. SVA can also induce complete autophagy processes and promote self-replication through the PERK and ATF6 pathways [30]. Our findings align with these reports and propose a new possible mechanism for how SVA promotes autophagy in porcine cells to increase the rate of viral infection. Studies have shown that transfecting cells with plasmids encoding viral 2A and 2B proteins significantly decreased the expression of LC3 protein via a mechanism involving MARCHF8 [19]; exactly how SVA affects LC3 proteins is not yet clear. The circ_8521/miR-324/*LC3A* regulatory axis identified in the present study provides new evidence for the fact that SVA promotes LC3 protein expression in porcine cells.

In eukaryotes, the LC3 protein family includes LC3A (two splicing variants), LC3B, LC3C, GABARAP, GABARAPL1, and GABARAPL2. LC3B is related to the development and maturation of autophagosomes and is also the most studied LC3 protein, which is often used to monitor autophagic activity. Despite the high level of homology between the LC3 family members, they appear to have unique functions [44]. To date, the molecular function, regulation, and cellular localization of LC3A have been poorly investigated. Nevertheless, a connection between LC3A expression patterns and cancer has been reported. For instance, the elevated expression of LC3A can enhance autophagy and mitochondrial metabolism, promoting the proliferation of lung cancer cells while weakening their invasiveness. Conversely, silencing LC3A reduces the proliferation of cancer cells but enhances their invasive characteristics [45]. In colorectal tumor cells, the “stone-like” intracellular structure (SLS) expression pattern of LC3A may represent abnormal or excessive autophagy, which is related to cancer cell metastasis and a poor prognosis [46]. The SLS expression pattern of LC3A is also strongly correlated with a poor outcome in non-small cell lung carcinoma [47], breast cancer [48], prostate cancer [49], skin squamous cell carcinoma [50], and brain malignancies [51]. As an oncolytic virus, SVA has shown anti-tumor efficacy in various cancers. SVA can enter cells by recognizing tumor endothelial marker 8, and in some cases, can cause cell death and stimulate anti-tumor effects by autophagy [52]; however, the specific mechanism of action remains unclear. It is worth noting that LC3A may be targeted by SVA to exert oncolytic effects. Further research is needed to determine whether SVA employs the same mechanisms in cancer and healthy cells.

**Figure 6:**
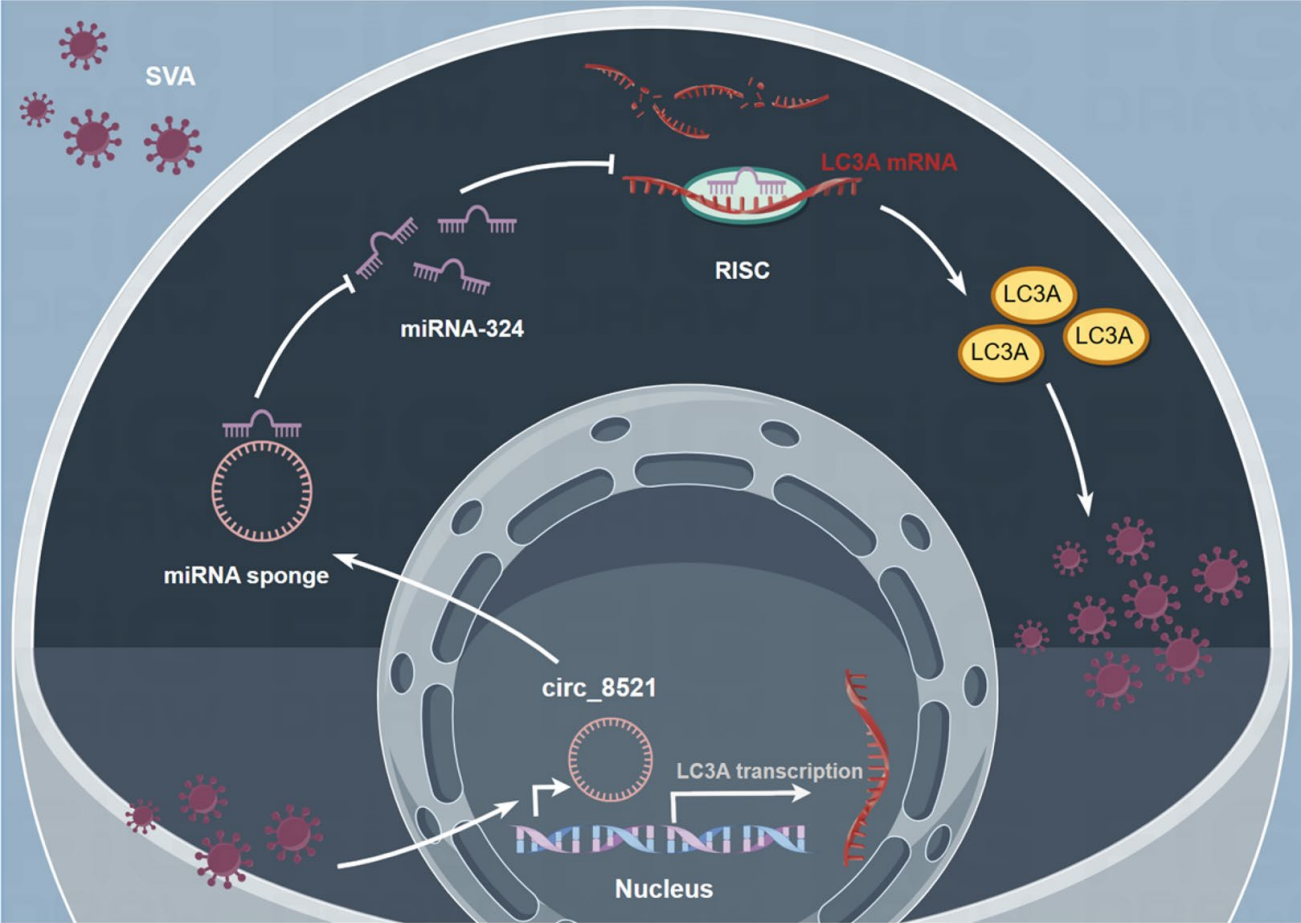
**The schematic diagram of circ_8521/miR-324/*LC3A*** **axis regulating SVA.** In SVA-infected cells, circ_8521, as an endogenous miR-324 sponge, inhibits the activity of miR-324 binding to *LC3A*, leading to increased expression of LC3A and subsequent viral replication.

In conclusion, the results of the present study revealed that SVA induced circ_8521 functioned as an endogenous miR-324 sponge to sequester miR-324, which promoted LC3A expression and ultimately SVA infection (Figure 6). Therefore, circ_8521 might be a potential therapeutic target to inhibit SVA infection. In addition, the role of circ_8521 expression and its miRNA sponging function should be explored in the context of viral infections other than SVA.

### Supplementary Information

**Additional file 1:** **Primers sequences used in this study.**

**Additional file 2: SVA induces upregulation of circ_8521 in primary porcine nasal mucosal epithelial cells and intestinal porcine epithelial cells. A.** The levels of circ_8521 in primary porcine nasal mucosal epithelial cells at 0, 3, 10.5, 18 h post-infection were determined by qRT-PCR. **B.** The levels of circ_8521 in intestinal porcine epithelial cells at 0, 3, 10.5, 18 h post-infection were determined by qRT-PCR.

**Additional file 3: Cell viability evaluation. A.** PK-15 cells were transfected with circ_8521 siRNA. NC siRNA served as a control. At 24 h post-transfection, cell viability was determined. **B.** PK-15 cells were transfected with pEZX-circ_8521. At 24 h post-transfection, cell viability was determined. **C.** PK-15 cells were transfected with miR-324 mimic. NC mimic served as a control. At 24 h post-transfection, cell viability was determined. **D.** PK-15 cells were transfected with miR-324 inhibitor. NC inhibitor served as a control. At 24 h post-transfection, cell viability was determined. **E**. PK-15 cells were transfected with *LC3A* siRNA. NC siRNA served as a control. At 24 h post-transfection, cell viability was determined.

**Additional file 4:** **circ_8521 regulated SVA infection in primary porcine nasal mucosal epithelial cells.** Primary porcine nasal mucosal epithelial cells were transfected with circ_8521 siRNA (50 nM) (**A–C**) or the circ_8521 overexpression plasmid pEZX-circ_8521 (0.5 ng) (**D–F**). NC siRNA served as a control. At 24 h post-transfection, cells were infected with SVA for 18 h. The effect of circ-8521 on SVA infection was subsequently analyzed. **A**. qRT-PCR analysis circ_8521 levels. **B.** qRT-PCR analysis SVA VP1 mRNA levels. **C**. Western blotting analysis of SVA protein expression. **D.** qRT-PCR analysis of circ_8521 levels. **E.** qRT-PCR analysis SVA VP1 mRNA levels. **F.** Western blotting analysis of SVA protein expression. **P < 0.01; ***P < 0.001.

**Additional file 5:** **circ_8521 regulated SVA infection in intestinal porcine epithelial cells.** Intestinal porcine epithelial cells were transfected with circ_8521 siRNA (50 nM) (**A–C**) or the circ_8521 overexpression plasmid pEZX-circ_8521 (0.5 ng) (**D–F**). NC siRNA served as a control. At 24 h post-transfection, cells were infected with SVA for 18 h. The effect of circ-8521 on SVA infection was subsequently analyzed. **A.** qRT-PCR analysis circ_8521 levels. B. qRT-PCR analysis SVA VP1 mRNA levels. **C.** Western blotting analysis of SVA protein expression. **D.** qRT-PCR analysis of circ_8521 levels. E. qRT-PCR analysis SVA VP1 mRNA levels. F. Western blotting analysis of SVA protein expression. **P < 0.01; ****P < 0.0001.

**Additional file 6: SVA increases autophagy levels in cells via the circ_8521/miR-324 axis. A.** PK-15 cells were transfected with circ_8521 siRNA. NC siRNA served as a control. At 24 h post-transfection, the cells were infected. LC3-I, LC3-II, and p62 expression was examined by Western blotting. **B.** PK-15 cells were transfected with an miR-324 mimic. NC mimic served as a control. At 24 h post-transfection, the cells were infected. LC3-I, LC3-II, and p62 expression was examined by Western blotting. **C.** PK-15 cells were transfected with an miR-324 inhibitor. NC inhibitor served as a control. At 24 h post-transfection, the cells were infected. LC3-I, LC3-II, and p62 expression was examined by Western blotting.

## Data Availability

RNA-Seq data were deposited into the Gene Expression Omnibus database under accession number GSE231770. The datasets used and analysed during the current study are available from the corresponding author on reasonable request.

## References

[1] Segales J, Barcellos D, Alfieri A, Burrough E, Marthaler D (2017). Senecavirus A. Vet Pathol.

[2] Adams MJ, Lefkowitz EJ, King AM, Bamford DH, Breitbart M, Davison AJ, Ghabrial SA, Gorbalenya AE, Knowles NJ, Krell P, Lavigne R, Prangishvili D, Sanfaçon H, Siddell SG, Simmonds P, Carstens EB (2015). Ratification vote on taxonomic proposals to the International Committee on Taxonomy of Viruses (2015). Arch Virol.

[3] Willcocks MM, Locker N, Gomwalk Z, Royall E, Bakhshesh M, Belsham GJ, Idamakanti N, Burroughs KD, Reddy PS, Hallenbeck PL, Roberts LO (2011). Structural features of the Seneca Valley virus internal ribosome entry site (IRES) element: a picornavirus with a pestivirus-like IRES. J Virol.

[4] Yang X, Hu Z, Fan S, Zhang Q, Zhong Y, Guo D, Qin Y, Chen M (2018). Picornavirus 2A protease regulates stress granule formation to facilitate viral translation. PLoS Pathog.

[5] Zhang X, Zhu Z, Yang F, Cao W, Tian H, Zhang K, Zheng H, Liu X (2018). Review of Seneca Valley virus: a call for increased surveillance and research. Front Microbiol.

[6] Liu F, Wang Q, Huang Y, Wang N, Shan H (2020). A 5-year review of Senecavirus A in China since its emergence in 2015. Front Vet Sci.

[7] Oliveira TES, Michelazzo MMZ, Fernandes T, de Oliveira AG, Leme RA, Alfieri AF, Alfieri AA, Headley SA (2017). Histopathological, immunohistochemical, and ultrastructural evidence of spontaneous Senecavirus A-induced lesions at the choroid plexus of newborn piglets. Sci Rep.

[8] Sun P, Zhang S, Qin X, Chang X, Cui X, Li H, Zhang S, Gao H, Wang P, Zhang Z, Luo J, Li Z (2018). Foot-and-mouth disease virus capsid protein VP2 activates the cellular EIF2S1-ATF4 pathway and induces autophagy via HSPB1. Autophagy.

[9] Zhang J, Zhang H, Sun W, Jiao C, Xiao P, Han J, Nan F, Xie C, Ha Z, Li Z, Xie Y, Meng Y, Lu H, Jin N (2021). Genetic evolution and epidemiological analysis of Seneca Valley virus (SVV) in China. Virus Res.

[10] Vannucci FA, Linhares DC, Barcellos DE, Lam HC, Collins J, Marthaler D (2015). Identification and complete genome of Seneca Valley virus in vesicular fluid and sera of pigs affected with idiopathic vesicular disease, Brazil. Transbound Emerg Dis.

[11] Leme RA, Oliveira TE, Alcântara BK, Headley SA, Alfieri AF, Yang M, Alfieri AA (2016). Clinical manifestations of Senecavirus A infection in neonatal pigs, Brazil, 2015. Emerg Infect Dis.

[12] Hause BM, Myers O, Duff J, Hesse RA (2016). Senecavirus A in pigs, United States, 2015. Emerg Infect Dis.

[13] Xu W, Hole K, Goolia M, Pickering B, Salo T, Lung O, Nfon C (2017). Genome wide analysis of the evolution of Senecavirus A from swine clinical material and assembly yard environmental samples. PLoS ONE.

[14] Sun D, Vannucci F, Knutson TP, Corzo C, Marthaler DG (2017). Emergence and whole-genome sequence of Senecavirus A in Colombia. Transbound Emerg Dis.

[15] Wu Q, Zhao X, Bai Y, Sun B, Xie Q, Ma J (2017). The first identification and complete genome of Senecavirus A affecting pig with idiopathic vesicular disease in China. Transbound Emerg Dis.

[16] Saeng-Chuto K, Rodtian P, Temeeyasen G, Wegner M, Nilubol D (2018). The first detection of Senecavirus A in pigs in Thailand, 2016. Transbound Emerg Dis.

[17] Arzt J, Bertram MR, Vu LT, Pauszek SJ, Hartwig EJ, Smoliga GR, Palinski R, Stenfeldt C, Fish IH, Hoang BH, Phuong NT, Hung VV, Vu PP, Dung NK, Dong PV, Tien NN, Dung DH (2019). First detection and genome sequence of senecavirus A in Vietnam. Microbiol Resour Announc.

[18] Zhao K, Zhang S, Liu X, Guo X, Guo Z, Zhang X, Yuan W (2022). The game between host antiviral innate immunity and immune evasion strategies of senecavirus A—a cell biological perspective. Front Immunol.

[19] Sun D, Kong N, Dong S, Chen X, Qin W, Wang H, Jiao Y, Zhai H, Li L, Gao F, Yu L, Zheng H, Tong W, Yu H, Zhang W, Tong G, Shan T (2022). 2AB protein of Senecavirus A antagonizes selective autophagy and type I interferon production by degrading LC3 and MARCHF8. Autophagy.

[20] Li H, Lin C, Qi W, Sun Z, Xie Z, Jia W, Ning Z (2023). Senecavirus A-induced glycolysis facilitates virus replication by promoting lactate production that attenuates the interaction between MAVS and RIG-I. PLoS Pathog.

[21] Fernandes MHV, Maggioli MF, Otta J, Joshi LR, Lawson S, Diel DG (2019). Senecavirus A 3C protease mediates host cell apoptosis late in infection. Front Immunol.

[22] Qian S, Fan W, Liu T, Wu M, Zhang H, Cui X, Zhou Y, Hu J, Wei S, Chen H, Li X, Qian P (2017). Seneca valley virus suppresses host type I interferon production by targeting adaptor proteins MAVS, TRIF, and TANK for cleavage. J Virol.

[23] Levine B, Mizushima N, Virgin HW (2011). Autophagy in immunity and inflammation. Nature.

[24] Levine B, Kroemer G (2008). Autophagy in the pathogenesis of disease. Cell.

[25] Mao J, Lin E, He L, Yu J, Tan P, Zhou Y (2019). Autophagy and viral infection. Adv Exp Med Biol.

[26] Mohamud Y, Shi J, Qu J, Poon T, Xue YC, Deng H, Zhang J, Luo H (2018). Enteroviral infection inhibits autophagic flux via disruption of the SNARE complex to enhance viral replication. Cell Rep.

[27] Ait-Goughoulte M, Kanda T, Meyer K, Ryerse JS, Ray RB, Ray R (2008). Hepatitis C virus genotype 1a growth and induction of autophagy. J Virol.

[28] Dreux M, Chisari FV (2009). Autophagy proteins promote hepatitis C virus replication. Autophagy.

[29] Liang Q, Luo Z, Zeng J, Chen W, Foo SS, Lee SA, Ge J, Wang S, Goldman SA, Zlokovic BV, Zhao Z, Jung JU (2016). Zika virus NS4A and NS4B proteins deregulate Akt-mTOR signaling in human fetal neural stem cells to inhibit neurogenesis and induce autophagy. Cell Stem Cell.

[30] Hou L, Dong J, Zhu S, Yuan F, Wei L, Wang J, Quan R, Chu J, Wang D, Jiang H, Xi Y, Li Z, Song H, Guo Y, Lv M, Liu J (2019). Seneca valley virus activates autophagy through the PERK and ATF6 UPR pathways. Virology.

[31] Yang L, Wilusz JE, Chen LL (2022). Biogenesis and regulatory roles of circular RNAs. Annu Rev Cell Dev Biol.

[32] Zhou WY, Cai ZR, Liu J, Wang DS, Ju HQ, Xu RH (2020). Circular RNA: metabolism, functions and interactions with proteins. Mol Cancer.

[33] Mo Y, Liu Y, Lu A, Zhang H, Tang L (2021). Role of circRNAs in viral infection and their significance for diagnosis and treatment (review). Int J Mol Med.

[34] Zhao T, Zheng Y, Hao D, Jin X, Luo Q, Guo Y, Li D, Xi W, Xu Y, Chen Y, Gao Z, Zhang Y (2019). Blood circRNAs as biomarkers for the diagnosis of community-acquired pneumonia. J Cell Biochem.

[35] miRanda. http://www.bioinformatics.com.cn/local_miranda_miRNA_target_prediction_120. Accessed 25 Sep 2022.

[36] Zhu M, Cai Y, Zhao W, He C, Yang Y, Gao Q, Su S (2020). Long non-coding RNAs are associated with Seneca Valley virus infection. Vet Microbiol.

[37] Tang X, Zhang R, Gao L, Lv X, Sun Y, Ma J (2023). LncRNA 8244-ssc-miR-320-CCR7 regulates IFN-β during SVA infecting PK-15 cells. Microorganisms.

[38] Wang C, Chen Y, Yang X, Du Y, Xu Z, Zhou Y, Yang X, Wang X, Zhang C, Li S, Yang Y, Li W, Liu X (2023). The porcine piRNA transcriptome response to Senecavirus a infection. Front Vet Sci.

[39] Niu D, Wu Y, Lian J (2023). Circular RNA vaccine in disease prevention and treatment. Signal Transduct Target Ther.

[40] Liu X, Zhang Y, Zhou S, Dain L, Mei L, Zhu G (2022). Circular RNA: An emerging frontier in RNA therapeutic targets, RNA therapeutics, and mRNA vaccines. J Control Release.

[41] Qu L, Yi Z, Shen Y, Lin L, Chen F, Xu Y, Wu Z, Tang H, Zhang X, Tian F, Wang C, Xiao X, Dong X, Guo L, Lu S, Yang C, Tang C, Yang Y, Yu W, Wang J, Zhou Y, Huang Q, Yisimayi A, Liu S, Huang W, Cao Y, Wang Y, Zhou Z, Peng X, Wang J, Xie XS, Wei W (2022). Circular RNA vaccines against SARS-CoV-2 and emerging variants. Cell.

[42] Song J, Hou L, Quan R, Wang D, Jiang H, Liu J (2022). Synergetic contributions of viral VP1, VP3, and 3C to activation of the AKT-AMPK-MAPK-MTOR signaling pathway for Seneca valley virus-induced autophagy. J Virol.

[43] Wen W, Li X, Yin M, Wang H, Qin L, Li H, Liu W, Zhao Z, Zhao Q, Chen H, Hu J, Qian P (2021). Selective autophagy receptor SQSTM1/ p62 inhibits Seneca Valley virus replication by targeting viral VP1 and VP3. Autophagy.

[44] Schaaf MB, Keulers TG, Vooijs MA, Rouschop KM (2016). LC3/GABARAP family proteins: autophagy-(un)related functions. Faseb J.

[45] Miao CC, Hwang W, Chu LY, Yang LH, Ha CT, Chen PY, Kuo MH, Lin SC, Yang YY, Chuang SE, Yu CC, Pan ST, Kao MC, Chang CR, Chou YT (2022). LC3A-mediated autophagy regulates lung cancer cell plasticity. Autophagy.

[46] Giatromanolaki A, Koukourakis MI, Harris AL, Polychronidis A, Gatter KC, Sivridis E (2010). Prognostic relevance of light chain 3 (LC3A) autophagy patterns in colorectal adenocarcinomas. J Clin Pathol.

[47] Karpathiou G, Sivridis E, Koukourakis MI, Mikroulis D, Bouros D, Froudarakis ME, Giatromanolaki A (2011). Light-chain 3A autophagic activity and prognostic significance in non-small cell lung carcinomas. Chest.

[48] Sivridis E, Koukourakis MI, Zois CE, Ledaki I, Ferguson DJ, Harris AL, Gatter KC, Giatromanolaki A (2010). LC3A-positive light microscopy detected patterns of autophagy and prognosis in operable breast carcinomas. Am J Pathol.

[49] Giatromanolaki A, Sivridis E, Mendrinos S, Koutsopoulos AV, Koukourakis MI (2014). Autophagy proteins in prostate cancer: relation with anaerobic metabolism and Gleason score. Urol Oncol.

[50] Sivridis E, Giatromanolaki A, Karpathiou G, Karpouzis A, Kouskoukis C, Koukourakis MI (2011). LC3A-positive "stone-like" structures in cutaneous squamous cell carcinomas. Am J Dermatopathol.

[51] Giatromanolaki A, Sivridis E, Mitrakas A, Kalamida D, Zois CE, Haider S, Piperidou C, Pappa A, Gatter KC, Harris AL, Koukourakis MI (2014). Autophagy and lysosomal related protein expression patterns in human glioblastoma. Cancer Biol Ther.

[52] Luo D, Wang H, Wang Q, Liang W, Liu B, Xue D, Yang Y, Ma B (2022). Senecavirus A as an oncolytic virus: prospects, challenges and development directions. Front Oncol.

