# Supplementary figures and images for: circRNA_8521 promotes Senecavirus A infection by sponging miRNA-324 to regulate LC3A
Source: Vet Res. 2024 Apr 5;55:43. doi: 10.1186/s13567-024-01291-0 (PMC10996121; doi:10.1186/s13567-024-01291-0)

## Slide 1
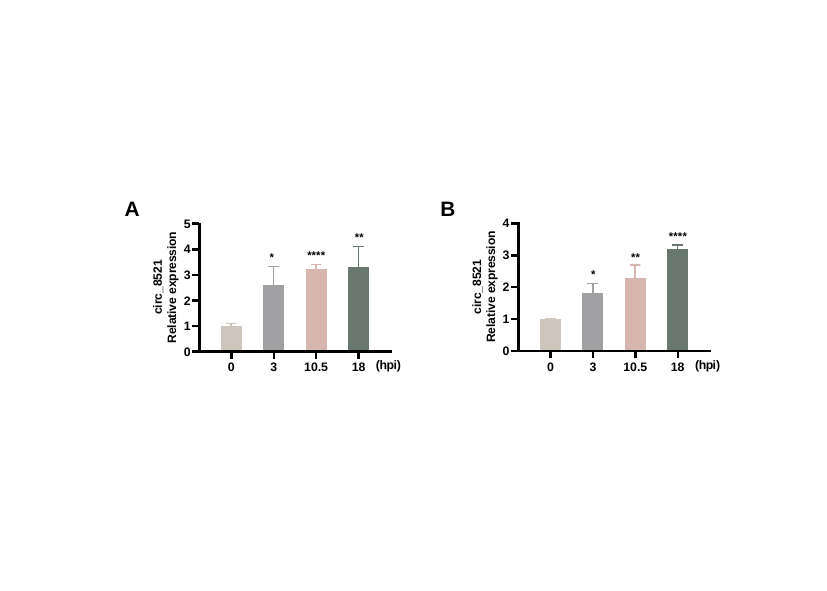

B
A

Supplement: Supplementary file 2 — Additional file 2: SVA induces upregulation of circ_8521 in primary porcine nasal mucosal epithelial cells and intestinal porcine epithelial cells. A. The levels of circ_8521 in primary porcine nasal mucosal epithelial cells at 0, 3, 10.5, 18 h post-infection were determined by qRT-PCR. B. The levels of circ_8521 in intestinal porcine epithelial cells at 0, 3, 10.5, 18 h post-infection were determined by qRT-PCR. [file 13567_2024_1291_MOESM2_ESM.pptx]

## Slide 1
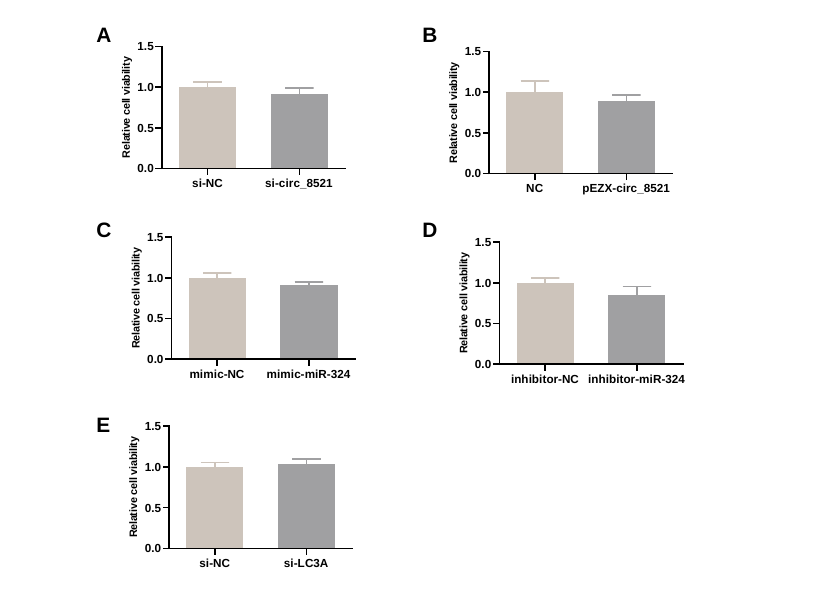

A
B
C
D
E

Supplement: Supplementary file 3 — Additional file 3: Cell viability evaluation. A. PK-15 cells were transfected with circ_8521 siRNA. NC siRNA served as a control. At 24 h post-transfection, cell viability was determined. B. PK-15 cells were transfected with pEZX-circ_8521. At 24 h post-transfection, cell viability was determined. C. PK-15 cells were transfected with miR-324 mimic. NC mimic served as a control. At 24 h post-transfection, cell viability was determined. D. PK-15 cells were transfected with miR-324 inhibitor. NC inhibitor served as a control. At 24 h post-transfection, cell viability was determined. E. PK-15 cells were transfected with LC3A siRNA. NC siRNA served as a control. At 24 h post-transfection, cell viability was determined. [file 13567_2024_1291_MOESM3_ESM.pptx]

## Slide 1
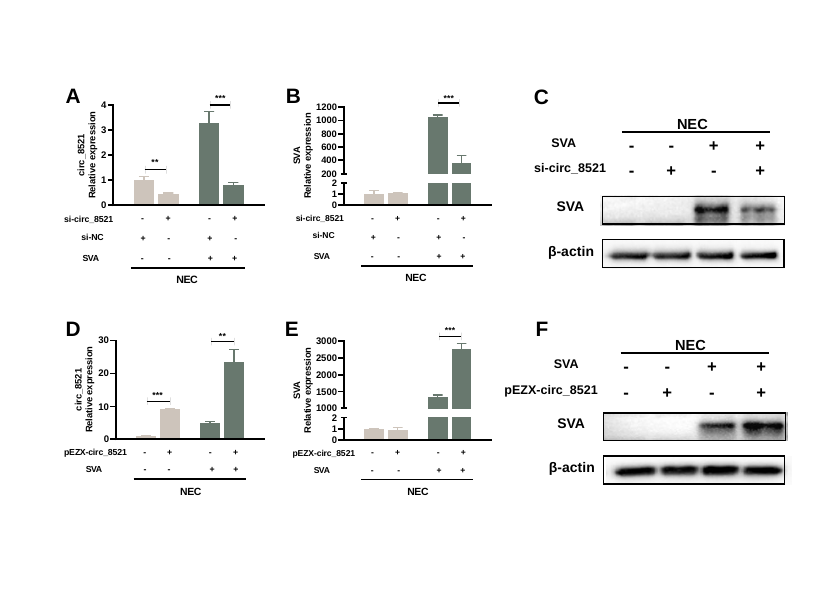

B
A
C
NEC
| | | SVA | - | - | + | + |
| --- | --- | --- | --- | --- | --- | --- |
| si-circ\_8521 | | si-circ\_8521 | - | + | - | + |
SVA
β-actin
F
D
E
NEC
| | | SVA | - | - | + | + |
| --- | --- | --- | --- | --- | --- | --- |
| pEZX-circ\_8521 | | si-circ\_8521 | - | + | - | + |
SVA
β-actin

Supplement: Supplementary file 4 — Additional file 4: circ_8521 regulated SVA infection in primary porcine nasal mucosal epithelial cells. Primary porcine nasal mucosal epithelial cells were transfected with circ_8521 siRNA (50 nM) (A–C) or the circ_8521 overexpression plasmid pEZX-circ_8521 (0.5 ng) (D–F). NC siRNA served as a control. At 24 h post-transfection, cells were infected with SVA for 18 h. The effect of circ-8521 on SVA infection was subsequently analyzed. A. qRT-PCR analysis circ_8521 levels. B. qRT-PCR analysis SVA VP1 mRNA levels. C. Western blotting analysis of SVA protein expression. D. qRT-PCR analysis of circ_8521 levels. E. qRT-PCR analysis SVA VP1 mRNA levels. F. Western blotting analysis of SVA protein expression. **P < 0.01; ***P < 0.001. [file 13567_2024_1291_MOESM4_ESM.pptx]

## Slide 1
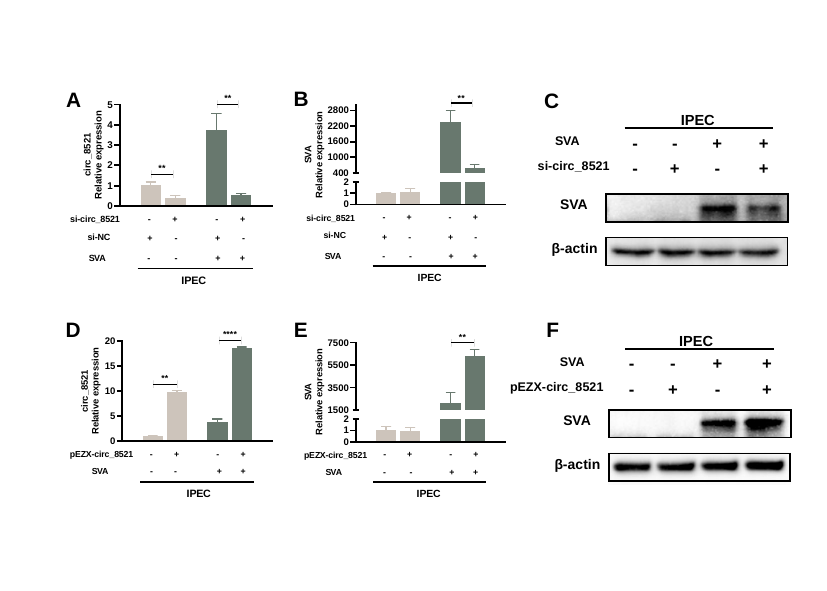

B
A
C
IPEC
| | | SVA | - | - | + | + |
| --- | --- | --- | --- | --- | --- | --- |
| si-circ\_8521 | | si-circ\_8521 | - | + | - | + |
SVA
β-actin
D
E
F
IPEC
| | | SVA | - | - | + | + |
| --- | --- | --- | --- | --- | --- | --- |
| pEZX-circ\_8521 | | si-circ\_8521 | - | + | - | + |
SVA
β-actin

Supplement: Supplementary file 5 — Additional file 5: circ_8521 regulated SVA infection in intestinal porcine epithelial cells. Intestinal porcine epithelial cells were transfected with circ_8521 siRNA (50 nM) (A–C) or the circ_8521 overexpression plasmid pEZX-circ_8521 (0.5 ng) (D–F). NC siRNA served as a control. At 24 h post-transfection, cells were infected with SVA for 18 h. The effect of circ-8521 on SVA infection was subsequently analyzed. A. qRT-PCR analysis circ_8521 levels. B. qRT-PCR analysis SVA VP1 mRNA levels. C. Western blotting analysis of SVA protein expression. D. qRT-PCR analysis of circ_8521 levels. E. qRT-PCR analysis SVA VP1 mRNA levels. F. Western blotting analysis of SVA protein expression. **P < 0.01; ****P < 0.0001. [file 13567_2024_1291_MOESM5_ESM.pptx]

## Slide 1
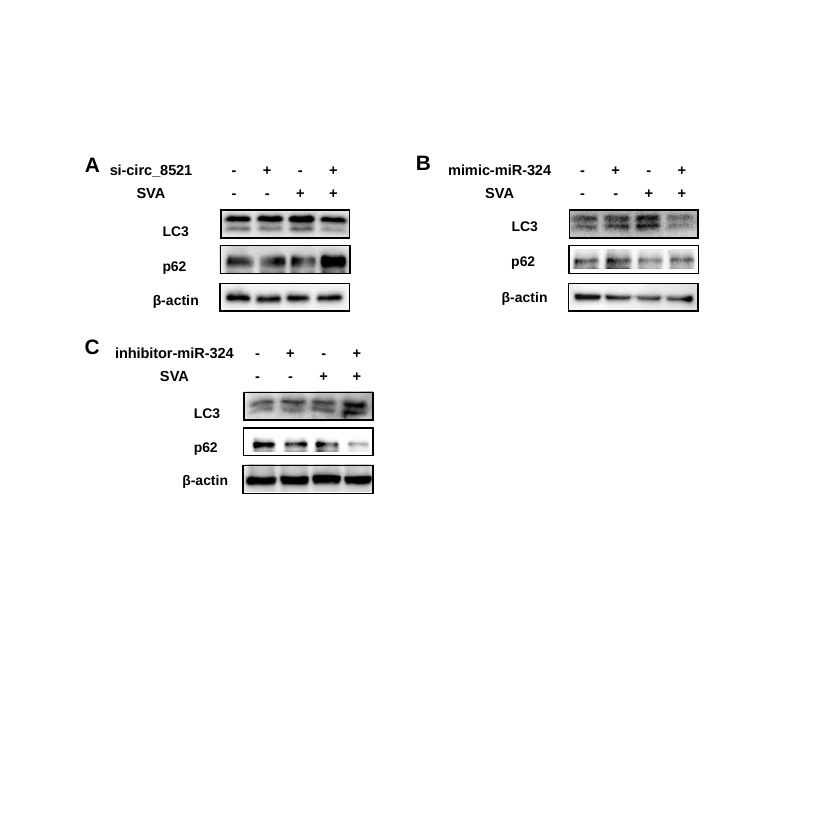

B
A
| si-circ\_8521 | - | + | - | + |
| --- | --- | --- | --- | --- |
| SVA | - | - | + | + |
| mimic-miR-324 | - | + | - | + |
| --- | --- | --- | --- | --- |
| SVA | - | - | + | + |
LC3
LC3
p62
p62
β-actin
β-actin
C
| inhibitor-miR-324 | - | + | - | + |
| --- | --- | --- | --- | --- |
| SVA | - | - | + | + |
LC3
p62
β-actin

Supplement: Supplementary file 6 — Additional file 6: SVA increases autophagy levels in cells via the circ_8521/miR-324 axis. A. PK-15 cells were transfected with circ_8521 siRNA. NC siRNA served as a control. At 24 h post-transfection, the cells were infected. LC3-I, LC3-II, and p62 expression was examined by Western blotting. B. PK-15 cells were transfected with an miR-324 mimic. NC mimic served as a control. At 24 h post-transfection, the cells were infected. LC3-I, LC3-II, and p62 expression was examined by Western blotting. C. PK-15 cells were transfected with an miR-324 inhibitor. NC inhibitor served as a control. At 24 h post-transfection, the cells were infected. LC3-I, LC3-II, and p62 expression was examined by Western blotting. [file 13567_2024_1291_MOESM6_ESM.pptx]
